# Mode of delivery and the risk of lymphoblastic leukemia during childhood—A Swedish population‐based cohort study

**DOI:** 10.1002/ijc.70027

**Published:** 2025-07-04

**Authors:** Christina‐Evmorfia Kampitsi, Hanna Mogensen, Mats Heyman, Maria Feychting, Giorgio Tettamanti

**Affiliations:** ^1^ Unit of Epidemiology Institute of Environmental Medicine, Karolinska Institutet Stockholm Sweden; ^2^ Department of Women's and Children's Health Childhood Cancer Research Unit, Karolinska Institutet Stockholm Sweden; ^3^ Department of Pediatric Oncology Karolinska University Hospital Stockholm Sweden; ^4^ Department of Molecular Medicine and Surgery Karolinska Institutet Stockholm Sweden

**Keywords:** cesarean section, child, leukemia, leukemia, B‐cell, leukemia, lymphoid

## Abstract

Cesarean section (CS) rates have been increasing beyond medically warranted thresholds, despite potential long‐term adverse outcomes. Previous research on CS delivery and childhood leukemia is conflicting but suggests an increased acute lymphoblastic leukemia (ALL) risk in children delivered by planned CS. It has been suggested that maternal and pregnancy conditions predisposing to pregnancy complications might confound such an association; therefore, we aimed to elucidate the relationship between delivery mode and ALL in Swedish children. To this end, we studied all children born in Sweden between 1982–1989 and 1999–2014, when comprehensive information on delivery mode was available (*n* = 2,442,330). Pregnancy conditions, delivery mode, and childhood ALL diagnoses (<20 years) were retrieved from nationwide registers. Cox proportional hazards regression was used to assess the association between delivery mode and childhood ALL, adjusting for maternal and pregnancy conditions. We observed an increased ALL risk among children delivered by planned CS (HR = 1.21, 95% CI 0.96–1.54), driven by B‐cell precursor ALL (HR = 1.29, 95% CI 1.01–1.67). The associations were concentrated among boys and at peak ages of ALL incidence (≤5 years) and persisted after accounting for potential confounders, including maternal and perinatal factors. Unplanned CS was not associated with increased risk of childhood ALL. Our nationwide study supports an association between planned CS and an increased B‐cell precursor ALL risk in Swedish children, irrespective of maternal and pregnancy conditions. Possible underlying mechanisms, such as lack of exposure to maternal vaginal microbiota or decreased stress hormones at birth, require further exploration.

## What's New?

Infants born by cesarean section (CS) are at increased risk of certain long‐term adverse health outcomes, including childhood leukemia. The influence of maternal factors, pregnancy, and perinatal conditions on these associations, however, remains unclear. In this study of more than 2 million children born in Sweden, the authors examined relationships between delivery mode and risk of childhood acute lymphoblastic leukemia (ALL). Analysis of data from nationwide registers reveals that planned, but not unplanned, CS is associated with an increased risk of childhood ALL, independent of maternal and perinatal factors. Further investigation is needed to elucidate biological mechanisms underlying this association.

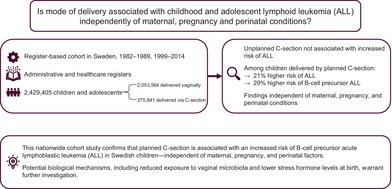

**Abbreviations**
ALL: acute lymphoblastic leukemia
AML: acute myeloid leukemia
BMI: body mass index
CI: confidence interval
CS: cesarean section
HR: hazard ratio
MBR: Medical Birth Register

## 1 INTRODUCTION

Rates of cesarean section (CS) are rising globally,^1^ with a median rate of 25.2% reported in 31 European countries, albeit with substantial regional disparities.^2^, ^3^ Sweden has also experienced a notable but comparably moderate rise in CS rates, from 5% in the early 1970s to 18.6% in 2021.^4^, ^5^ When medically indicated, CS is associated with a reduction in maternal and/or fetal mortality and morbidity.^6^ However, the World Health Organization has historically stressed that “there is no justification for any region to have a CS rate higher than 10%–15%”,^7^ with subsequent studies confirming that CS rates over 9%–15% are not associated with further reductions in mortality outcomes.^8^ Given the discrepancy between recommendations and the growing rate of CS, understanding the potential long‐term effects of delivery by CS on the offspring is becoming increasingly important.^9^

Delivery by CS has been linked to increased risk for several long‐term immune‐related adverse outcomes, including asthma,^10^, ^11^ allergies,^12^, ^13^ and type I diabetes mellitus.^14^ Childhood leukemia, the most prevalent type of cancer during childhood, has also been suggested to originate from immune system development and abnormal immune responses to infections.^15^ As such, several researchers have investigated the relationship between delivery by CS and leukemia risk, with conflicting results.^16^ Where an increased risk was found, it was only for acute lymphoblastic leukemia (ALL) rather than acute myeloid leukemia (AML); additionally, studies that differentiated between planned and unplanned CS found increased risks only for the former.

Several mechanisms have been proposed to explain a potentially increased childhood ALL risk following planned rather than unplanned CS, including altered bacterial communities in the newborn^17^, ^18^ and decreased levels of stress hormones at birth.^19^ This association, however, could instead be due to confounding by indication, as delivery by CS may be a marker for maternal and pregnancy conditions, such as maternal diabetes^20^, ^21^ or birth defects in the offspring,^22^ which predispose both to pregnancy complications and to a higher risk of childhood ALL. Few studies have taken such conditions into account. Thus, the aim of this work was to elucidate the relationship between mode of delivery and the risk of ALL in a large, nationwide cohort of Swedish children and adolescents with complete information on pregnancy and delivery conditions.

## 2 MATERIALS AND METHODS

### 2.1 Study design, population, and data sources

**FIGURE 1:**
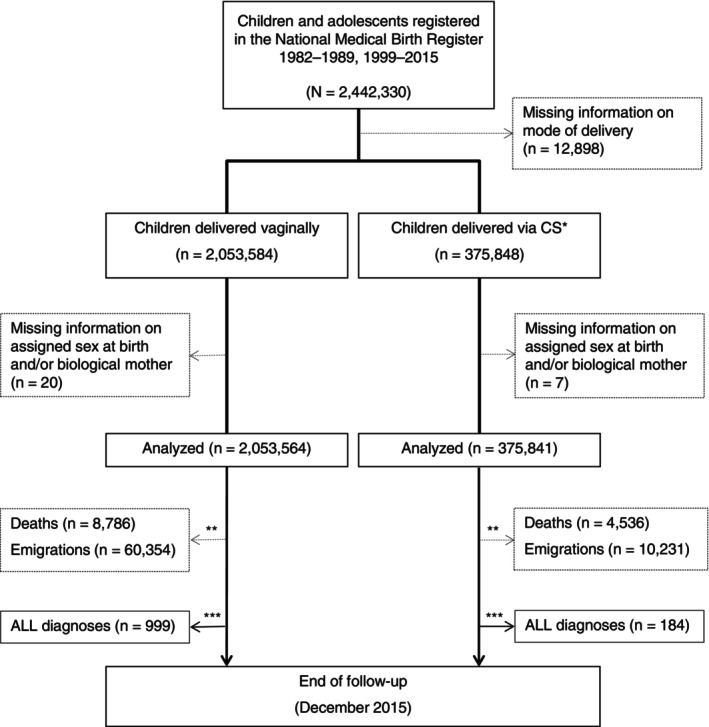
Cohort flow chart. *Including pre‐labor (planned and acute) and post‐labor CS. **Prior to any ALL diagnosis. ***Retrieved through linkage with the National Cancer Register. ALL, acute lymphoblastic leukemia; CS, cesarean section.

We conducted a population‐based cohort study that included children registered in the Swedish National Medical Birth Register (MBR) between 1982–1989 and 1999–2015 (*n* = 2,442,330, Figure 1). Established in 1973, the MBR records information on nearly all deliveries in Sweden,^23^ including standardized reports of maternal and fetal health conditions detected during perinatal, delivery, or neonatal care. The overall quality of the MBR is high. Common maternal diagnoses, such as gestational diabetes and pre‐eclampsia, are considered to be of good validity, while most birth and neonatal factors have excellent validity; the validity of infant diagnoses is comparable to corresponding information in the National Patient Register.^23^ The period of the study was selected to enable access to comprehensive information on mode of delivery. During this period, we had the ability to differentiate between CS occurring before and after labor onset—and further distinguish pre‐labor CS into planned and acute. This distinction may be crucial, both due to the differences in early microbial colonization and hormonal stress response between planned and unplanned CS, and because the clinical indications for each differ—introducing distinct confounding structures. Aside from absolute indications like abnormal fetal presentation, planned CS is more common in the context of maternal characteristics such as advanced age, obesity, diabetes, preeclampsia, or fetal characteristics such as macrosomia or congenital anomalies.^24^ Unplanned CS typically arises from emergent complications during labor, including fetal distress or failure to progress.^25^ Children with missing information on mode of delivery, biological mother, or assigned sex at birth were excluded from the study (*n* = 12,925; 0.5%, Figure 1).

The study population was linked to the National Cancer Register via the unique personal identity number^26^ in order to obtain information on childhood and adolescent cancer diagnoses (<20 years of age). Reporting to the National Cancer Register is compulsory since 1958, ensuring high validity and completeness; approximately 99% of tumors are morphologically verified, while a validation study has found underreporting to be less than 4%.^27^ We then used information from the National Cancer Register to classify childhood leukemia diagnoses according to the International Classification of Childhood Cancer, third edition (ICCC‐3).^28^ Information on the immunophenotype of children with ALL (B‐cell precursor, T‐cell) and their genotype (the most abundant subtypes, i.e., high hyperdiploidy‐HeH or ETV6::RUNX1, or other) was retrieved from the Swedish Childhood Cancer Register,^29^ which originated in the early 1980s. Genotypic information was only available from 1992 onwards, however. Children enrolled in this study were followed from birth until the first of the following events: cancer diagnosis, death, emigration, 20th birthday, or end of follow‐up (December 2015). Information on emigrations and deaths was retrieved from the Total Population Register^30^ and the National Cause of Death Register,^31^ respectively.

### 2.2 Covariates

Perinatal characteristics and conditions were retrieved from the MBR and included birth date, assigned sex at birth (male/female), healthcare region of residence at birth (six regions), birth weight by gestational age (categorized as small, appropriate, and large, using the 10th and 90th percentiles of birth weight distribution in the population, according to sex, birth decade, and gestational age), and diagnosis of any birth defect. Information on certain maternal characteristics and conditions was also obtained from the MBR and includes age at delivery (categorized as ≤25, 25–29, 30–34, 35–39, and ≥ 40 years), reported infections during pregnancy, and diagnoses of pre‐eclampsia or diabetes (pregestational and gestational). We were further able to calculate the maternal body mass index (BMI) by using information on height (self‐reported) and weight (usually measured at registration to antenatal care and therefore closely approximating pre‐pregnancy BMI). Finally, we retrieved information on the mothers' attained educational level at the child's birth (primary, secondary, post‐secondary) from the national censuses and the Longitudinal integrated database for health insurance and labor market studies.^32^

### 2.3 Statistical analysis

Cox proportional hazards regression models were used to estimate hazard ratios (HR) and corresponding 95% confidence intervals (CI) of childhood ALL, B‐cell precursor ALL, and the most prevalent B‐cell precursor ALL subtypes (HeH/ ETV6::RUNX1), comparing children delivered by CS to those delivered vaginally. The analysis was repeated to distinguish between pre‐ and post‐labor CS, with the former further dichotomized into planned and acute pre‐labor CS. Analyses were adjusted for offspring sex, period of birth, birth weight by gestational age, region of residence at birth, and any birth defect, as well as maternal age, education, preeclampsia, diabetes, and infections during pregnancy. The analyses were repeated stratifying by offspring sex and age at leukemia diagnosis. As very few adolescents diagnosed with leukemia at ages 16–19 had been delivered by CS, we only examined the diagnostic age groups of 0–5, 6–10, and 11–15 years of age in the age‐stratified analysis. Finally, we further adjusted for maternal BMI in a sensitivity analysis. While maternal height and weight were recorded throughout the study period, their level of missingness varies by decade, ranging from 4% to 24% (100% for weight during 1990–1991).^23^ Data were prepared with SAS 9.4, whereas all analyses were performed with STATA 14.2.

## 3 RESULTS

Among the 2,429,405 participants included in this study, we identified 375,841 (15.5%) children delivered by CS (Figure 1). Of those, 213,306 were reported as delivered by a CS occurring before the onset of labor (including 136,342 planned). Birth and maternal characteristics of the participants are reported in Table 1. Children delivered by CS were more likely to be born small or large for gestational age, or with a birth defect, compared to children delivered vaginally. Moreover, a higher proportion among them were born to older mothers, overweight or obese mothers, or mothers with conditions such as diabetes mellitus and pre‐eclampsia.

**TABLE 1:** Baseline characteristics of the study cohort (Swedish children and adolescents, born 1982–1989 and 1999–2015), by mode of delivery.

	Delivered vaginally *n* (%)	Delivered by CS^a^ *n* (%)
*Offspring characteristics and conditions*		
Sex		
Male	1,050,171 (51.1)	199,095 (53.0)
Female	1,003,393 (48.9)	176,746 (47.0)
Birth weight by gestational age		
SGA	198,866 (9.7)	48,973 (13.0)
AGA	1,652,654 (80.6)	277,553 (74.0)
LGA	198,878 (9.7)	48,713 (13.0)
Birth defect, any	72,410 (3.5)	21,199 (5.6)
*Maternal characteristics and conditions during pregnancy*		
Maternal age (years)		
≤25	522,126 (25.4)	63,304 (16.8)
25–29	565,023 (27.5)	87,068 (23.2)
30–34	636,104 (31.0)	126,218 (33.6)
35–39	279,435 (13.6)	77,840 (20.7)
≥40	50,876 (2.5)	21,411 (5.7)
Maternal education		
Primary	220,666 (10.8)	38,701 (10.4)
Secondary	876,395 (43.1)	156,531 (42.0)
Post‐secondary	938,806 (46.1)	177,756 (47.6)
Maternal diabetes	27,461 (1.3)	13,372 (3.6)
Maternal pre‐eclampsia	45,537 (2.2)	27,479 (7.3)
Maternal infections	9192 (0.5)	2852 (0.8)
Maternal overweight and obesity	494,003 (28.9)	122,952 (34.4)

Abbreviations: AGA, appropriate for gestational age; CS, cesarean section; LGA, large for gestational age; SGA, small for gestational age.

^a^ Including pre‐labor (planned and acute) and post‐labor CS.

In the study population, we identified 1495 children with leukemia, including 1183 children with ALL (of whom 952 were diagnosed with B‐cell precursor ALL). The overall HR for ALL in children born by CS, compared to vaginal births, was 1.07 (95% CI 0.91–1.25) (Table 2). The risk of ALL was more pronounced in children delivered by planned CS occurring before labor onset (HR 1.21; 95% CI, 0.96–1.54), especially for B‐cell precursor ALL (HR 1.29; 95% CI, 1.01–1.67). Results for planned pre‐labor CS were similar after adjustment for maternal and pregnancy conditions, with only small attenuations observed (Table 2). Results also remained largely consistent in a sensitivity analysis where we further adjusted for maternal BMI (Supplementary Table 1). It is worth noting that any observed attenuations in this analysis were primarily due to the more restricted population, rather than solely the adjustment for maternal BMI (as within the study population restricted to those with information on maternal BMI, results remained consistent regardless of whether maternal BMI was taken into account during the adjustment process). When stratifying by assigned sex at birth, an association between planned pre‐labor CS and B‐cell precursor ALL was observed among boys (HR_adjusted_ 1.41; 95% CI, 1.00–1.99), but not girls (HR_adjusted_ 1.09; 95% CI, 0.74–1.60) (Supplementary Table 2). Analyses stratifying by age at leukemia diagnosis revealed that the increased ALL risk in planned pre‐labor CS was concentrated among children diagnosed at ages 0–5 (HR_adjusted_ for ALL 1.37; 95% CI 1.05–1.78; HR_adjusted_ for B‐cell precursor ALL 1.35; 95% CI 1.02–1.80) (Table 3). Risk estimates were more pronounced among children with the most prevalent subtypes, namely ETV6::RUNX1 rearrangement and HeH, as indicated in Supplementary Table 3. This pattern held true for children diagnosed with B‐cell precursor ALL at any age (HR_adjusted_ 1.40; 95% CI, 0.96–2.03) and for those diagnosed up to 5 years of age (HR_adjusted_ 1.57; 95% CI, 1.00–2.33).

**TABLE 2:** Mode of delivery and the risk of acute lymphoblastic leukemia in Swedish children and adolescents, born 1982–1989 and 1999–2015.

Mode of delivery	No. of ALL cases	Crude HR (95% CI)	No. of ALL cases	Adjusted HR (95% CI)
*ALL*				
Vaginal	999	1.00 (ref)	995	1.00 (ref)
CS	184	1.07 (0.91–1.25)	182	1.05 (0.89–1.23)
Pre‐labor	106	1.05 (0.86–1.29)	105	1.03 (0.84–1.27)
Planned	74	1.21 (0.96–1.54)	74	1.19 (0.94–1.51)
Acute	30	0.90 (0.63–1.29)	29	0.89 (0.61–1.29)
Post‐labor	78	1.09 (0.86–1.37)	77	1.07 (0.85–1.36)
*B‐cell precursor ALL*				
Vaginal	804	1.00 (ref)	802	1.00 (ref)
CS	148	1.07 (0.89–1.27)	147	1.04 (0.87–1.25)
Pre‐labor	84	1.04 (0.83–1.30)	84	1.02 (0.81–1.28)
Planned	64	1.29 (1.01–1.67)	64	1.25 (0.97–1.62)
Acute	18	0.68 (0.43–1.08)	18	0.71 (0.44–1.14)
Post‐labor	63	1.11 (0.86–1.43)	63	1.07 (0.83–1.39)

*Note*: Adjusted model includes: Offspring sex, period of birth, birth weight by gestational age, region of residence at birth, any birth defect. Maternal age, education, preeclampsia, diabetes, infections. Numbers do not add up because of missing values.

Abbreviations: ALL, acute lymphoblastic leukemia; CI, confidence interval; CS, cesarean section; HR, hazards ratio.

**TABLE 3:** Association between mode of delivery and acute lymphoblastic leukemia in Swedish children and adolescents, born 1982–1989 and 1999–2015—depending on age at diagnosis.

	Diagnosed 0–5 years		Diagnosed 6–10 years		Diagnosed 11–15 years	
Mode of delivery	No. of ALL cases	HR (95% CI)	No. of ALL cases	HR (95% CI)	No. of ALL cases	HR (95% CI)
*ALL*						
Vaginal	701	1.00 (ref)	183	1.00 (ref)	79	1.00 (ref)
CS	138	1.10 (0.92–1.33)	31	0.94 (0.63–1.40)	11	0.86 (0.44–1.68)
Pre‐labor	79	1.11 (0.88–1.40)	17	1.01 (0.61–1.52)	8	0.91 (0.42–1.98)
Planned	62	1.37 (1.05–1.78)	9	0.92 (0.46–1.83)	<5	0.72 (0.22–2.37)
Acute	16	0.76 (0.46–1.26)	7	1.21 (0.56–2.64)	5	1.39 (0.52–3.73)
Post‐labor	59	1.10 (0.84–1.44)	14	0.86 (0.48–1.52)	<5	0.74 (0.22–2.47)
*B‐cell precursor ALL*						
Vaginal	598	1.00 (ref)	137	1.00 (ref)	56	1.00 (ref)
CS	116	1.09 (0.89–1.33)	20	0.80 (0.49–1.30)	10	1.18 (0.57–2.43)
Pre‐labor	66	1.08 (0.84–1.40)	11	0.88 (0.47–1.65)	7	1.13 (0.48–2.63)
Planned	53	1.35 (1.02–1.80)	8	1.07 (0.51–2.25)	<5	0.99 (0.30–3.28)
Acute	12	0.68 (0.38–1.21)	<5	0.52 (0.13–2.13)	<5	1.52 (0.48–4.80)
Post‐labor	50	1.10 (0.82–1.47)	9	0.71 (0.35–1.44)	<5	1.31 (0.37–4.58)

*Note:* Reported model includes: Offspring sex, period of birth, birth weight by gestational age, region of residence at birth, any birth defect. Maternal age, education, preeclampsia, diabetes, infections. Numbers do not add up because of missing values.

Abbreviations: ALL, acute lymphoblastic leukemia; CI, confidence interval; CS, cesarean section; HR, hazards ratio.

## 4 DISCUSSION

In this population‐based cohort study of almost 2.5 million children, we found an increased ALL risk in children delivered by planned CS, stemming from B‐cell precursor ALL, and specifically the HeH/ ETV6::RUNX1 subtypes. This heightened risk was observed primarily among boys. Further, rather than persisting throughout the age range, it was concentrated in children diagnosed with ALL up to 5 years of age. The associations were not explained by maternal and pregnancy conditions, or offspring perinatal factors; in fact, adjusted and unadjusted estimates were largely similar, supporting the notion that confounding by indication does not explain the increased ALL risk associated with planned CS. Unplanned CS was not associated with the risk of childhood ALL at any age.

Our findings align with several prior reports indicating a positive association between CS and risk of childhood ALL. A recent meta‐analysis of 16 studies found that children delivered by planned CS had an 18% increased risk of developing ALL compared to those born vaginally.^33^ Notably, studies that differentiated between age at leukemia diagnosis also found this increased risk to be concentrated in young children, particularly those aged 0–3 years^34^ or 2–4 years.^35^ Additionally, studies that could distinguish ALL immunophenotype noted a particularly elevated risk for B‐lineage ALL^34^; yet, no further stratification based on the underlying genotype has been performed, to the best of our knowledge. Moreover, only one previous study, conducted on children from Minnesota, performed a sex‐stratified analysis—albeit without the ability to differentiate between planned and unplanned CS.^36^ Contrary to our findings of an increased risk of ALL among boys delivered by planned CS, that study reported a higher ALL risk primarily in girls delivered by any CS, possibly influenced by an excess of planned CS procedures during female deliveries as noted by the authors. In contrast, our observation of an increased ALL risk among boys delivered by planned CS aligns with the previously reported higher incidence of ALL among boys.^37^ Previous studies have not been able to consistently differentiate between planned and unplanned CS. Moreover, they often employed a case–control design and relied on self‐reported CS data. Perhaps most importantly, they have not been able to fully account for maternal, pregnancy, and perinatal conditions that could impact both the mode of delivery and childhood ALL risk—therefore confounding the association between the two.

Various mechanisms have been postulated to account for the increased risk of childhood ALL arising from planned but not unplanned CS. First, although infants born by unplanned CS with ruptured amniotic sac are exposed to the microbial diversity present in the birth canal, those born by planned CS acquire skin and hospital environment microbiota instead—independent of prophylactic antibiotics administered before CS.^17^, ^38^ This difference in early microbial colonization could affect immune system development, increasing susceptibility to immune‐related diseases, including ALL. Second, the absence of the hormonal stress response to labor during planned CS occurring before labor onset may also contribute to an increased susceptibility to ALL.^19^ Labor triggers the release of hormones, including cortisol and the catecholamines noradrenaline and adrenaline.^19^, ^39^, ^40^, ^41^ While infants born by unplanned CS and vaginal delivery show comparable levels of these stress hormones,^39^ those born by planned CS before labor onset show markedly decreased concentrations.^40^ These hormones are known to regulate the immune response and may eliminate leukaemic and preleukemic cells arising in utero; in their absence, immune system development and function may be impacted, increasing the risk of childhood ALL.^42^ Future research should explore such potential links between planned CS and immune system development in early life; such investigations can provide valuable insights into the complex etiology of childhood leukemia.

To our knowledge, this population‐based study is the first to examine delivery by CS and childhood ALL risk while comprehensively accounting for maternal, pregnancy, and offspring conditions that may influence the association. By considering these factors, we were able to effectively control for potential confounding by indication. Additionally, our study benefits from a large sample size, a long‐term follow‐up, and the ability to differentiate between planned and unplanned CS. Finally, the register‐based design enabled us to access prospectively collected, objective, and standardized information on both mode of delivery and ALL diagnoses. This approach served to minimize the potential for measurement error and information bias.

Despite the strengths of our study, it is important to acknowledge certain limitations when interpreting the results. While the register‐based design enabled us to access comprehensive data on delivery mode, maternal and pregnancy conditions, and ALL diagnoses, it also restricted us to capturing only formal diagnoses of maternal conditions. As such, we may have missed less severe cases. Moreover, it should be noted that stratified analyses to explore potential patterns by sex and age were exploratory and not supported by formal tests of interaction; as such, any observed subgroup differences should be interpreted with caution. Additionally, our study was conducted in Sweden, where the CS rate is relatively low (11%–17% during the study period^5^) and adheres closely to the WHO guidelines. This, coupled with the rarity of childhood ALL, resulted in limited statistical power, particularly in analyses stratified by leukaemic genotype, sex or age at ALL diagnosis; age‐stratified analyses were especially constrained by the small number of cases in older age groups, which may limit the precision of those estimates. It is worth noting that other countries have much higher CS rates, sometimes approaching 50% of all births,^2^ and this disparity might affect the transportability of our findings. However, this would only be the case if the distribution of potential effect modifiers differs between populations. Lastly, even though we expect that the association between CS and offspring ALL will persist across populations, the impact may vary depending on CS rates.

In conclusion, in this extensive population‐based cohort study, we have confirmed a relationship between planned CS and an increased risk of childhood B‐cell precursor ALL. The increased risk seemed to be driven by associations found among boys and at peak ages of ALL incidence (up to 5 years of age). Notably, this association was not driven by maternal or pregnancy‐related factors or offspring perinatal conditions. Further research is warranted to clarify the underlying mechanisms and gain a deeper understanding of the etiology of childhood ALL. Finally, the study findings add to the growing body of evidence suggesting the need to reexamine non‐medically warranted CS in light of adverse long‐term outcomes for the offspring.

## AUTHOR CONTRIBUTIONS

**Christina‐Evmorfia Kampitsi:** Conceptualization; methodology; software; formal analysis; writing – original draft; writing – review and editing; visualization. **Hanna Mogensen:** Data curation; writing – review and editing. **Mats Heyman:** Data curation; writing – review and editing. **Maria Feychting:** Conceptualization; methodology; project administration; supervision; funding acquisition; writing – review and editing. **Giorgio Tettamanti:** Conceptualization; methodology; writing – review and editing; supervision; data curation.

## FUNDING INFORMATION

This project was funded by grants from the Swedish Research Council and the Swedish Cancer Society. The research process was independent of the funders.

## CONFLICT OF INTEREST STATEMENT

The authors declare no conflict of interest.

## ETHICS STATEMENT

Ethical approval for the study was granted by the Regional Ethical Review Board, Stockholm, Sweden (Dnr 2011/634‐31/4; and amendment Dnr 2016/27‐32).

## Supporting information


**Data S1:** Supporting Information

## Data Availability

Swedish laws and regulations do not allow sharing of personal sensitive data, which can only be made available for researchers who fulfill legal requirements for access to personal sensitive data. Eligible individuals can apply for the data from the National Board of Health and Welfare in Sweden (https://bestalladata.socialstyrelsen.se/) and from Statistics Sweden (https://www.scb.se/vara-tjanster/bestall-data-och-statistik/). Further information is available from the corresponding author upon request.
