# Supplementary material for: Mode of delivery and the risk of lymphoblastic leukemia during childhood—A Swedish population‐based cohort study
Source: Int J Cancer. 2025 Jul 4;157(10):2041–8. doi: 10.1002/ijc.70027 (PMC12439074; doi:10.1002/ijc.70027)
Supplement: Supplementary file 1 — Data S1: Supporting Information [file IJC-157-2041-s001.pdf]

Mode of delivery and the risk of lymphoblastic leukemia during childhood—a Swedish population-based cohort study.

Christina-Evmorfia Kampitsi, Hanna Mogensen, Mats Heyman, Maria Feychting, Giorgio Tettamanti

SUPPLEMENTARY MATERIAL CONTENTS

Supplementary Table 1. Association between mode of delivery and acute lymphoblastic leukemia in Swedish children and adolescents born 1982–1989 and 1999–2015—further adjusted for maternal BMI.

Supplementary Table 2. Mode of delivery and the risk of acute lymphoblastic leukemia in Swedish children and adolescents, born 1982–1989 and 1999–2015—stratified by assigned sex at birth.

Supplementary Table 3. Mode of delivery and the risk of B-cell precursor acute lymphoblastic leukemia with high hyperdiploidy or ETV6-RUNX1 rearrangement in Swedish children and adolescents, born 1982–1989 and 1999–2015.

Supplementary Table 1. Association between mode of delivery and acute lymphoblastic leukemia in Swedish children and adolescents born 1982–1989 and 1999–2015—further adjusted for maternal BMI.

Mode of delivery	No. of ALL cases	Model 1 HR (95% CI)	Model 2 HR (95% CI)
ALL			
**Vaginal**	815	1.00 (ref)	1.00 (ref)
**CS**	148	1.06 (0.89–1.27)	1.06 (0.89–1.27)
**Pre-labor**	87	1.07 (0.86–1.34)	1.07 (0.86–1.34)
**Planned**	60	1.17 (0.90–1.53)	1.17 (0.90–1.52)
**Acute**	25	1.05 (0.70–1.57)	1.05 (0.70–1.57)
**Post-labor**	61	1.05 (0.81–1.37)	1.05 (0.81–1.37)
B-cell precursor ALL			
**Vaginal**	669	1.00 (ref)	1.00 (ref)
**CS**	120	1.04 (0.85–1.27)	1.03 (0.85–1.26)
**Pre-labor**	69	1.03 (0.80–1.33)	1.02 (0.80–1.32)
**Planned**	51	1.19 (0.89–1.59)	1.19 (0.89–1.58)
**Acute**	16	0.84 (0.51–1.39)	0.84 (0.51–1.39)
**Post-labor**	51	1.05 (0.78–1.40)	1.04 (0.78–1.39)

Model 1 includes:
Offspring sex, period of birth, birth weight by gestational age, region of residence at birth, any birth defect
Maternal age, education, preeclampsia, diabetes, infections

Model 2 additionally includes maternal BMI

Abbreviations:
HR , hazards ratio ; CI , confidence interval ; CS , cesarean section ;
ALL , acute lymphoblastic leukemia ; BMI , body mass index

Numbers do not add up because of missing values

Supplementary Table 2. Mode of delivery and the risk of acute lymphoblastic leukemia in Swedish children and adolescents, born 1982–1989 and 1999–2015—stratified by assigned sex at birth.

Mode of delivery	No. of ALL cases	Boys HR (95% CI)	No. of ALL cases	Girls HR (95% CI)
ALL				
**Vaginal**	531	1.00 (ref)	464	1.00 (ref)
**CS**	107	1.14 (0.92–1.40)	75	0.95 (0.74–1.21)
**Pre-labor**	55	1.01 (0.77–1.34)	50	1.04 (0.78–1.41)
**Planned**	41	1.26 (0.91–1.73)	33	1.12 (0.78–1.60)
**Acute**	14	0.77 (0.45–1.32)	15	1.03 (0.61–1.73)
**Post-labor**	52	1.31 (0.98–1.75)	25	0.79 (0.53–1.18)
B-cell precursor ALL				
**Vaginal**	407	1.00 (ref)	395	1.00 (ref)
**CS**	86	1.18 (0.93–1.50)	61	0.89 (0.68–1.17)
**Pre-labor**	43	1.03 (0.75–1.41)	41	1.01 (0.73–1.39)
**Planned**	36	1.41 (1.00–1.99)	28	1.09 (0.74–1.60)
**Acute**	7	0.52 (0.24–1.09)	11	0.93 (0.50–1.70)
**Post-labor**	43	1.40 (1.02–1.92)	20	0.72 (0.46–1.13)

Reported model includes:
Period of birth, birth weight by gestational age, region of residence at birth, any birth defect
Maternal age, education, preeclampsia, diabetes, infections

Abbreviations:
HR , hazards ratio ; CI , confidence interval ; CS , cesarean section ; ALL , acute lymphoblastic leukemia

Numbers do not add up because of missing values

Supplementary Table 3. Mode of delivery and the risk of B-cell precursor acute lymphoblastic leukemia with high hyperdiploidy or ETV6-RUNX1 rearrangement in Swedish children and adolescents, born 1982–1989 and 1999–2015.

	Diagnosed at any age		Diagnosed 0–5 years	
**Mode of delivery**	**No. of ALL cases**	**HR (95% CI)**	**No. of ALL cases**	**HR (95% CI)**
**Vaginal**	329	1.00 (ref)	257	1.00 (ref)
**CS**	69	1.10 (0.85–1.44)	61	1.23 (0.92–1.63)
**Pre-labor**	36	1.12 (0.79–1.58)	33	1.31 (0.91–1.89)
**Planned**	31	1.40 (0.96–2.03)	28	1.57 (1.00–2.33)
**Acute**	5	0.78 (0.32–1.91)	5	1.10 (0.46–2.76)
**Post-labor**	33	1.09 (0.76–1.56)	28	1.14 (0.77–1.69)

Reported model includes:
Offspring sex, period of birth, birth weight by gestational age, region of residence at birth, any birth defect
Maternal age, education, preeclampsia, diabetes, infections

Abbreviations:
HR , hazards ratio ; CI , confidence interval ; CS , cesarean section

Numbers do not add up because of missing values
